# Metabolic Modifications by Common Respiratory Viruses and Their Potential as New Antiviral Targets

**DOI:** 10.3390/v13102068

**Published:** 2021-10-14

**Authors:** Jens Kleinehr, Janine J. Wilden, Yvonne Boergeling, Stephan Ludwig, Eike R. Hrincius

**Affiliations:** 1 Institute of Virology Muenster (IVM), Westfaelische Wilhelms-University Muenster, Von-Esmarch-Str. 56, D-48149 Muenster, Germany; j_klei45@uni-muenster.de (J.K.); janine.wilden@wwu.de (J.J.W.); borgelin@uni-muenster.de (Y.B.); ludwigs@uni-muenster.de (S.L.); 2 Cells in Motion Interfaculty Centre (CiMIC), Westfaelische Wilhelms-University Muenster, Von-Esmarch-Str. 56, D-48149 Muenster, Germany

**Keywords:** respiratory viruses, metabolism, host targeted antivirals

## Abstract

Respiratory viruses are known to be the most frequent causative mediators of lung infections in humans, bearing significant impact on the host cell signaling machinery due to their host-dependency for efficient replication. Certain cellular functions are actively induced by respiratory viruses for their own benefit. This includes metabolic pathways such as glycolysis, fatty acid synthesis (FAS) and the tricarboxylic acid (TCA) cycle, among others, which are modified during viral infections. Here, we summarize the current knowledge of metabolic pathway modifications mediated by the acute respiratory viruses respiratory syncytial virus (RSV), rhinovirus (RV), influenza virus (IV), parainfluenza virus (PIV), coronavirus (CoV) and adenovirus (AdV), and highlight potential targets and compounds for therapeutic approaches.

## 1. Introduction

Viral respiratory infections can spread rapidly within populations all around the globe. Their ability to circulate seasonally leads to recurring annual epidemics that cause mild symptoms or severe morbidity and even death. Susceptible groups usually comprise the elderly, immunocompromised persons and those with metabolic disorders, as well as—depending on the virus—children. The recent emergence of viral lung infections by SARS-CoV-2 and the resulting disease coronavirus disease 2019 (COVID-19) once again clearly showed the high risk of infection by acute respiratory viruses. The main reason for the hospitalizations of patients with severe respiratory viral infections is the development of a pulmonary inflammatory disorder, which is connected to lung tissue damage, edema and exacerbated inflammatory processes [1]. In general, the versatile aspects of the invading virus and the host, including strength of viral replication and viral spread, the expression of pathogenicity factors and the efficiency and manifestation of the immune response determine the severity of an infection and its symptoms. Some of the most common acute respiratory viruses are respiratory syncytial virus (RSV), rhinovirus (RV), influenza virus (IV), parainfluenza virus (PIV), coronavirus (CoV) and adenovirus (AdV). Each of these viruses has been linked to pneumonia, causing a significant disease burden [2].

**Figure 1:**
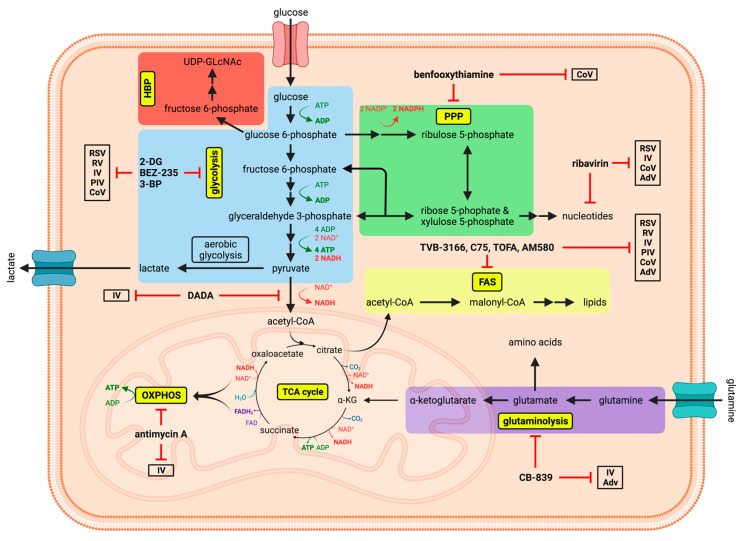
Schematic depiction of the main metabolic pathways, with potential compounds and their respective targets for antiviral metabolic interference. The graphic shows the dynamic network of some of the most important metabolic pathways (glycolysis, hexosamine biosynthetic pathway [HBP], pentose phosphate pathway [PPP], tricarboxylic acid [TCA] cycle, oxidative phosphorylation [OXPHOS], glutaminolysis and fatty acid synthesis [FAS]) in eukaryotic cells, which were shown to be altered and exploited during viral infections. Potential antiviral metabolic inhibitors are indicated along with those respiratory viruses they have been demonstrated to inhibit. Created with BioRender.com.

Since viruses, as obligate intracellular parasites, depend on the host cell, their propagation is based on the exploitation and consequentially the dysregulation of various cellular signaling pathways, which often have a wide range of function in important cellular processes like metabolism, proliferation and biosynthesis. Accordingly, viruses are able to modulate the host cell signaling machinery via blocking pattern recognition receptors [3], altering the antiviral immune response [4], impairing epithelial cell barrier function [5] and delaying programmed cell death to facilitate viral replication [6,7]. Some signaling pathways that are exploited by respiratory viruses and exhibit regulatory functions for their replication and/or pathogenicity have been extensively investigated, such as the mitogen-activated protein kinases (MAPKs) cascades [8,9,10,11], the nuclear factor kappa-light-chain-enhancer of activated B cells (NF-κB) signaling module [12,13,14,15,16] and metabolic mediators, such as the phosphatidylinositol 3-kinase (PI3K) pathway [17,18,19] and hypoxia inducible factor-1 (HIF-1) [20,21]. Among others, these cellular interfaces influence viral transport processes, viral replication and the generation of biological building blocks and energy for efficient virus propagation. Here, metabolic mediators like the PI3K signaling pathway represent important cellular modules, which are responsible for different cellular processes, including a special connection to metabolic reprogramming during viral infections [20,22,23,24]. Consequently, metabolic pathways are described to be altered in their activity during various viral infections of the respiratory tract. The main metabolic pathways, in this context, are glycolysis, which generates energy and fuels the tricarboxylic acid (TCA) cycle, glutaminolysis, which also fuels the TCA cycle and supports the synthesis of amino acids and nucleotides, the TCA cycle, which generates most electron donors for cellular respiration, fatty acid synthesis (FAS), that produces lipids, and the pentose phosphate pathway, which mainly generates nucleotides (Figure 1). Normally one or more pathways are upregulated while the activity of others can decrease during infections. As already reviewed by Sanchez et al. [25], the profile of cellular metabolic modifications differs from virus to virus but, in general, the production of new virus particles results in a higher demand for energy. Thus, an upregulation of pathways such as glycolysis or glutaminolysis can commonly be observed during infections. Furthermore, a higher demand for macromolecules such as fatty acids and nucleotides leads to alterations of the respective pathways that produce and modify these molecules [25].

Pharmacological manipulation of these crucial metabolic pathways, a process termed metabolic interference, may be suitable as a novel broad antiviral approach, since viruses appear to strongly depend on these metabolic alterations. Even though a lot is known about virus-induced changes of the host metabolism, there are, however, only a few treatments to counteract these effects that have been evaluated in clinical trials or even became licensed. The rebalancing of affected metabolic pathways to their basal levels of activity could represent a potential therapy to restore metabolic homeostasis in the infected host and may possibly mitigate viral spread and the severeness of infection.

Here, we summarize the impact of the afore-mentioned respiratory viruses RSV, RV, IV, PIV, CoV and AdV on the host metabolism and highlight metabolic interventions as possible therapeutic approaches (Figure 1).

## 2. Respiratory Syncytial Viruses

RSVs are enveloped, negative-sense single-stranded RNA [(-)ssRNA] viruses of the *Pneumoviridae* family. RSVs are the most prevalent viruses causing respiratory infections in infants, but also cause numerous cases among the immunocompromised and elderly [26,27,28,29]. In these groups RSV infections often lead to exacerbations of bronchiolitis, pneumonia and chronic obstructive pulmonary disease (COPD) [26,27,30,31] and, as already reviewed by Feldman et al. [32], may cause the development of asthma. Except for a prophylactically applicable neutralizing monoclonal antibody [33], there are currently no vaccines or effective medications against RSV available [34], even though an RNA interference-based therapy showed high tolerability and antiviral activity against RSV in a phase II clinical trial [35].

Upon RSV infection, human bronchial epithelial cells upregulate a large variety of genes. Among those are genes which are involved in various metabolic processes [36]. A recent study by Martín-Vicente et al. [34] using untargeted metabolomics discovered that RSV-infected carcinoma cells displayed increased levels of many metabolites of the central carbon metabolism, especially those of glycolysis, glutaminolysis and the TCA cycle, 12–18 h post infection (hpi). At later times of infection, the concentrations of amino acids, nucleotides and nucleoside di-/triphosphates were elevated, as well [34]. Morris et al. [20] observed that via the stabilization of HIF-1, an important metabolic regulator [37], RSV shifts the cell metabolism primarily from very oxygen-demanding pathways towards glycolysis and the PPP. Inhibition of the subunit HIF-1α not only reduced viral replication but also decreased levels of glycolytic enzymes [20]. In contrast to this metabolic shift, an in vivo study observed increased cellular respiration in lung fragments from RSV-infected mice [38]. Beside several glycolytic and PPP intermediates, Morris et al. also found elevated levels of glutamate, but not glutamine and α-ketoglutarate (α-KG), suggesting that glutamine is rather used for biosynthesis of amino acids and nucleotides instead of being oxidized in the TCA cycle. Unlike Martín-Vicente et al., Morris et al. did not see increased concentrations of most TCA cycle intermediates, which may be attributed to the fact that they used primary cells instead of carcinoma cells for their experiments [20]. However, both groups observed that the level of lactate was increased indicating that aerobic glycolysis (the Warburg effect [39,40]) was exploited [20,34]. Even though the Warburg effect seems to promote insufficient energy supply by uncoupling glycolysis from the TCA cycle, this type of metabolizing glucose was described to favor anabolic conditions in which biomass needs to be generated [41], e.g., during cell proliferation or viral replication. The fact that the inhibition of glucose metabolism by 2-deoxy-D-glucose (2-DG) or glucosamine reduced viral titers in vitro [42] further substantiates the significance of glucose for RSV replication. These observations suggest that the cells are shifted to an anabolic state to rapidly produce macromolecules such as amino acids, fatty acids and nucleotides, which are needed for virus reproduction [34]. Two metabolomics studies, analyzing urine metabolites in RSV-infected children in comparison with healthy controls, support the results of shifted metabolism due to RSV infection and report infection-specific metabolite levels of several of the afore-mentioned metabolic pathways [43,44]. These studies emphasize the potential existence of a defined urine metabolome of infected individuals compared with healthy controls with potential applications for a non-invasive diagnostic and the prediction of severeness of infection. Aside from urine samples others examined the metabolome of upper airway cells and fluids from nasal pharyngeal aspirates from RSV-infected infants (not yet peer-reviewed) [45]. Their data support several afore-mentioned results and strongly indicate that RSV is able to modify human metabolism. Interestingly, they observed that not only glycolysis and the TCA cycle was increased after infection but also that respiration almost reached its maximum while spare respiratory capacity diminished. Combined with lower levels of lactate in upper airway fluids, the data suggest that RSV infections feature only some aspects of the Warburg effect, while oxygen consumption and lactate levels tell a different story [45]. The partially varying results of different groups demonstrate how diverse metabolic reprogramming by viruses can be in various cell lines as well as between in vitro and in vivo experiments and that not only the type of virus, but also intrinsic cellular factors, determine the interplay of virus and metabolism.

Furthermore, in infected cells, amino sugars, nucleotide sugars and palmitic acid were more abundant compared with the levels observed in non-infected cells, allowing for ample post-translational protein modification, which is necessary for the maturation of several RSV proteins [34]. The levels of many lipids were deregulated by RSV, but, unlike palmitic acid, most lipids were downregulated with the onset of infection. The authors hypothesized that RSV might induce the degradation of lipids for the generation of energy and to reduce the cell’s control of its own redox state [34]. As lipids are involved in maintaining redox homeostasis [46], the theory seems plausible, since RSV was reported to increase oxidative stress and mitochondrial reactive oxygen species were described to support viral reproduction [47,48]. However, the role of lipids during RSV infections seems to be even more intriguing, since Ohol et al. [49] provided evidence that the inhibition of fatty acid synthase (FASN) reduced not only viral RNA and protein levels but, most distinctly, the amount of viral progeny and their infectivity in vitro and in vivo. The authors hypothesized that a changed composition of the viral envelope or an alteration of lipid rafts, which are important for viral polymerase activity, might explain their observations.

Taken together, RSV especially depends on aerobic glycolysis and a deregulated lipid metabolism to set the host cell in an anabolic state and to increase oxidative stress, thereby boosting its own reproduction capacity.

## 3. Rhinoviruses

RVs are small non-enveloped viruses with a positive-sense single-stranded RNA [(+)ssRNA] genome, belonging to the family of *Picornaviridae*. Human RVs are subdivided into three species (A, B and C) that contain more than 150 currently known serotypes [50]. In most cases, RV infects the upper respiratory tract and is a major cause of the common cold but can also infect the lower respiratory tract and has been associated with exacerbations of more severe clinical symptoms including asthma, bronchiolitis, pneumonia and COPD [26,30,51,52]. So far, there are no antiviral treatments or vaccines available.

A recent study by Gualdoni et al. has proven that RV induces a metabolic reprogramming to establish the optimal conditions for viral replication [17]. During the early infection of HeLa cells and primary human fibroblasts RV induces a PI3K-mediated increase in the expression of the glucose transporter GLUT1 to maximize the cellular uptake of glucose. The uptake of fatty acids, as well as the concentration of nucleotides, was also increased in HeLa cells. Mass spectrometry analysis suggested that RV-infected cells shift to an anabolic state by inducing glycogenolysis, lipogenesis and the PPP, while reducing fatty acid oxidation. Deprivation of the two important carbon sources glucose or glutamine, as well as the inhibition of glycolysis by 2-DG, impaired viral replication. Apparently, 2-DG prevented the shift to the anabolic state and even reduced viral replication in a murine in vivo model without eliciting obvious side effects [17].

Furthermore, other studies have shown that RV changes the whole lipidome towards an environment that is favorable for its own replication by influencing not only the number of certain types of lipids but also the length of their acyl carbon chains and their individual saturation [53]. Thus, it is not surprising that, among others, the inhibition of the FAS key enzyme FASN and the inhibition of ceramidase, an enzyme needed to produce sphingosines, clearly reduce RV genome replication, viral protein synthesis and the amount of infectious virus progeny [49,53]. Since RVs are non-enveloped viruses, their dependency on the lipid metabolism seems surprising, at first glance, and is highly intriguing. Data from Nguyen et al. [53] suggest that RV-induced lipidomic alterations occur early in infection and vary between early, intermediate and late phases of the viral life cycle. As it is common for picornaviruses and positive-strand RNA viruses in general, RV remodels intracellular membranes at the interface of the ER and Golgi to be used as replication organelles [54]. This remodeling mainly comprises increased levels of phosphatidylinositol 4-phosphate and cholesterol near RV replication sites and depends on various cellular proteins that modify and exchange lipids [54]. Most likely, the pattern of RV-induced lipidomic alterations is strongly connected to the amount and composition of these replication organelles. Other studies showed that RV infections trigger the translocation of the enzyme acid sphingomyelinase to the outer cellular membrane, where it causes the formation of ceramide- and glycosphingolipid-enriched domains that highly improve the uptake of RV particles [55,56]. Additionally, it can be speculated that RV influences various cellular signals, which are conveyed by different lipid molecules to maintain a stable environment during replication. As reviewed by Maceyka and Spiegel [57], sphingolipids, especially, have a broad range of signaling functions. High concentrations of ceramide are known to have a pro-apoptotic effect [58] and the deacylation of ceramide by ceramidase is the only known pathway for the generation of sphingosine [57]. With respect to the data from Nguyen et al., which showed reduced RV replication upon ceramidase inhibition [53], one might hypothesize that ceramidase not only produces important sphingosine but also prevents early apoptosis of the infected cell by counteracting to high intracellular levels of ceramide.

## 4. Influenza Viruses

IVs are members of the family of *Orthomyxoviridae* and are characterized by a lipid envelope and a (−)ssRNA genome which is fragmented into seven or eight segments. There are four types of IVs: A, B, C and D, among which influenza A and B virus (IAV and IBV) are the most prominent to cause infections in humans and the only ones that have been reported to elicit severe progress of disease in humans. Besides annually recurring epidemics of IAV and IBV, IAV has the potential to cause global pandemics, mostly due to its zoonotic property. Depending on the strain, IV can infect the upper and/or lower respiratory tract and in severe cases may lead to a systemic infection [59,60]. IV was reported to be associated with pneumonia, bronchitis and bronchiolitis and exacerbations of COPD [26,30,61]. Even though there are a few licensed drugs with a certain effectivity to counteract an infection, IV already developed resistances against most types of antivirals [62]. Although annually updated vaccines provide at least a partial protection from infection and severe morbidity, vaccination also faces limitations [63].

It has been known for some time that IV infections, especially IAV, depend on certain metabolites and alter the metabolism of infected cells. In the early 1950s, the importance of ample glucose for IV had already been demonstrated [64]. In 1961 it was reported for the first time that infection leads to an increase of glycolysis in embryonic chicken cells [65]. This has more recently been confirmed in Madin–Darby canine kidney cells [66] and primary normal human bronchial epithelial (NHBE) cells [22]. Glucose uptake in the lungs of infected patients was found to be increased, as well [22]. Moreover, an upregulation of glutaminolysis upon IAV infection was demonstrated [22,66]. Data from Smallwood et al. [22] indicate that infection-associated increases of PI3K/mechanistic target of rapamycin (mTOR) signaling and the expression of the transcription factor MYC (formerly c-MYC), a key regulator of glycolytic genes [67], are largely responsible for the reprogramming of glycolysis and glutaminolysis, which resulted in elevated viral titers [22]. In the same year another group also showed that the mTOR complexes 1 and 2 (mTORC1 and 2) are activated by IAV—mainly by its hemagglutinin protein—to boost viral protein production and replication [68]. Even though PI3K can also be activated by the detection of influenza vRNA [69], other studies proved the activation of PI3K via a direct interaction with the influenza non-structural protein 1 [70,71,72]. Given the fact that viral proteins can directly influence cellular regulatory proteins, this supports the assumption that metabolic alterations during infections can be triggered by specific interactions between viral components and host factors. Ritter et al. [66] described a distinct metabolic pattern for infected cells during the progression of infection with increased glucose uptake and higher levels of extracellular lactate and α-KG from 0–12 hpi. Between 12–24 hpi levels of various glycolytic intermediates were upregulated, while the concentration of adenosine triphosphate (ATP) decreased. Additionally, they observed a minor increase of several TCA cycle intermediates [66]. Their data suggest that while glycolysis is hyperactivated, glycolytic intermediates are used for the production of other molecules such as lipids, nucleotides and amino acids, which are necessary for large-scale virus production. Furthermore, the apparent induction of the Warburg effect, indicated by increased extracellular lactate, needs to be compensated by other agents to maintain TCA cycle homeostasis. Hence, the anaplerotic function of glutaminolysis could explain the increase of α-KG. Concurrently, Tian et al. [73] also observed an increase of glutamate in infected cells during the first viral replication cycle. This was put into context with an upregulated glutathione metabolism. By generating high amounts of oxidized coenzymes, a stable TCA cycle and, thus, energy supply is supposed to be maintained [73]. The value of glycolysis and glutaminolysis for IV replication is further demonstrated by the strongly reduced numbers of infected cells or lower viral titers respectively upon inhibition of mediators or crucial enzymes of both pathways [22,74,75,76]. The inhibition of mTORC1 and 2 by Torin1 or mTORC1 by rapamycin reduced the replication of IV [68]. By restricting IV-induced PI3K/mTOR pathway activation, thereby also reversing infection-mediated metabolic changes, the inhibitor BEZ235 was even efficient against IAV in an in vivo mouse model and led to reduced viral titers in their lungs, as well as improved survival rates of the animals [22]. In severe viral infections, morbidity and fatality are usually caused by the interplay of viral replication and the underlying immune response. Since IV vRNA-mediated activation of PI3K also stimulates interferon (IFN) induction [69], it can be speculated whether or not the use of BEZ235 and the consequential reduction of PI3K activity can also mitigate the antiviral IFN response. Even in this case, however, inhibiting PI3K/mTOR signaling resulted in an impairment of viral propagation and a beneficial outcome for the host. This is further indicated by a recent study declaring comparable results with a very similar PI3K inhibitor, called Pictilisib [77].

Another metabolically active compound, diisopropylamine dichloroacetate (DADA), inhibits pyruvate dehydrogenase kinase 4, which usually inactivates pyruvate dehydrogenase, hence promoting the uncoupling of glycolysis and the TCA cycle by favoring the conversion of pyruvate into lactate instead of acetyl-coenzyme A (acetyl-CoA). DADA treatment led to the decreased replication of IAV and improved survival of mice, accompanied by the suppression of cytokine storm and disorders of glucose and lipid metabolism in the blood [78]. A different study demonstrated that supplementation of ATP, after 24 h pretreatment with the glycolysis inhibitor 2-DG, could reverse the inhibitory effect on viral propagation in vitro [75], suggesting that not only the sufficient availability of glycolytic intermediates but also of energy-conveying molecules is of high importance in the context of IV replication and glycolysis. Besides, 2-DG and other molecules impair protein glycosylation, which, for example, reduces hemagglutinating activity [79]. In line with glycolytic metabolites being strongly redirected to the synthesis of building blocks for new viral progeny, an upregulation of PPP activity or PPP metabolites [22,66], the hexosamine biosynthetic pathway (HBP) [73] and FAS [22,73] was reported. The availability of nucleotides seems to be crucial for IAV replication since the metabolism of nucleotides and nucleosides, especially purine metabolism, was found to be highly increased during the viral replication cycle in A549 cells [73].

Just like the previously described viruses, IAV reprograms the lipid metabolism of its host cell to its own benefit [80]. Aside from a general induction of FAS, IAV displayed changes of the whole lipidome of infected cells in vitro and in vivo [81,82]. The importance of fatty acids, their modification and enhanced synthesis is emphasized by the fact that inhibiting FAS or the import of fatty acids leads to a clear reduction of IAV replication [83,84,85]. Triacsin C, an inhibitor of long chain fatty acid synthesis, also impaired replication of IAV, which was shown in a patent application (US2013190381A1) regarding this compound being suitable as a broad antiviral. Besides the utilization of fatty acids to generate energy, recent data suggest that IAV particularly depends on modifying lipids of the cellular membrane to facilitate budding and on palmitoylation of viral proteins, especially the surface protein hemagglutinin [85,86]. For IBV, a need for nucleoprotein palmitoylation was also observed, enabling intracellular trafficking [87].

Moreover, a mitochondrial proteomic profile favoring oxidative phosphorylation (OXPHOS) and an overall upregulation of OXPHOS activity was found in IAV-infected cells [22,88]. Consistently a strong reduction of IAV titers was observed when antimycin A, an inhibitor of complex III of the electron transport chain, was applied in vitro [89].

All these metabolic alterations show that IAV modifies the cellular carbon flux profoundly and transfers its host cell into an overall anabolic state, to allow for extensive virion production. The dimension of metabolic reprogramming by IV is further displayed by data showing altered immune cell activation and differentiation due to virus-induced metabolic changes and exacerbations of metabolic disorders (e.g., type 2 diabetes) that also constitute risk factors for infected individuals, which are extensively reviewed by Bahadoran et al. [90]. Furthermore, they summarize a wide array of immune response-metabolism correlations, which indicate that metabolic alterations during IV infections may partially be attributed to the induction of the innate immune response [90]. Several articles support such observations. For example, tumor necrosis factor-α increases glycolysis, ATP synthesis and lactate export, but reduces OXPHOS in epithelial cells [91]. Additionally, Chandler et al. [92] performed a cytokine-metabolome-wide association study with IAV-infected lungs of mice and found 396 metabolites, which were highly correlated with actions of inflammatory cytokines. IFNs have been discovered as common molecules to create an antiviral state in virus-infected cells [93]. As an example of the above-mentioned findings, aerobic glycolysis stimulates the expression of type II IFN-γ and high levels of glucose are required for IFN-γ synthesis [94,95]. Concurrently, type I IFNs stimulate glycolysis and glucose uptake [96]. Consequently, a fraction of altered metabolic activity upon virus infections can be caused by the host immune response.

## 5. Parainfluenza Viruses

PIVs are enveloped (−)ssRNA viruses of the family of *Paramyxoviridae*. In humans, four major subtypes exist, called human PIV (HPIV) 1–4. HPIVs usually infect the upper respiratory tract and spread to the lower airways during the progress of infection. Next to RSV, HPIV is the major reason for pediatric hospitalizations in connection with respiratory virus infections and is associated with various diseases such as otitis media, pharyngitis, conjunctivitis, croup, tracheobronchitis, pneumonia and the exacerbation of COPD [26,30,97]. Treatment against HPIV infection is restricted to immune system-supportive measures and, while research on potential vaccines (especially against HPIV3) is ongoing, there is currently no licensed vaccination [97,98].

Despite the role of HPIV in severe infections of children, elderly and immunocompromised, only few studies addressing its metabolic effects on the host cell are published. A clear reduction of HPIV3 titers was observed after treatment with the amino sugar glucosamine and especially 2-DG [42]. The inhibitory effect of 2-DG can presumably be accounted to the lack of glycolytic intermediates and/or ATP by glycolysis and was reversible by the addition of mannose, which can also drive glycolysis. Furthermore, the authors suggested that 2-DG, as well as glucosamine, caused the reduced or aberrant glycosylation of viral proteins, hence decreasing viral titers [42]. More recent data support the importance of glycosylation of viral proteins for HPIV propagation by showing reduced infectivity of HPIV3 after treatment with various inhibitors of α-glucosidase and α-mannosidase. This treatment impaired oligosaccharide processing on viral glycoproteins and reduced infectivity of progeny particles by reducing viral fusion activity [99].

Several reports deal with the impact of FAS on HPIV propagation. Since the composition of the host cell’s membrane affects the yield of viral progeny [100], it can be speculated whether HPIV modifies the lipidome of its host cell. Nevertheless, the synthesis of fatty acids is crucial for virus replication since the inhibition of FASN clearly impaired the production of infectious HPIV3 progeny in vitro with the strongest inhibitory effect at 72 hpi [49]. This is presumably the timepoint with the highest amount of budding viral progeny and thus the moment of greatest demand for lipids, which would explain the dependency of virion production on FAS.

Another crucial metabolite for HPIV is 5-hydroxytryptophan (5-HTP), the first downstream intermediate of the secondary tryptophan catabolic pathway. Even though no virus-induced upregulation of this pathway or of 5-HTP itself has been shown, its clear proviral effect can be demonstrated. Activation of the primary catabolic pathway by indoleamine 2,3-dioxygenase (IDO) depleted the tryptophan pool and, consequently, the 5-HTP pool and severely impaired HPIV replication [101]. Thus, the directed hyperactivation of IDO to deprive HPIV of 5-HTP may be considered as a potential treatment against HPIV.

## 6. Coronaviruses

CoVs are enveloped (+)ssRNA viruses and belong to the family of *Coronaviridae*. Aside from four species of human CoV (HCoV), which are steadily circulating in humans and cause usually mild, cold-like symptoms, three highly pathogenic species have appeared in the recent past: severe acute respiratory syndrome coronavirus (SARS-CoV) in 2002, middle east respiratory syndrome coronavirus (MERS-CoV) in 2012 and the current pandemic SARS-CoV-2, which emerged in December 2019 [102]. CoV infects the upper and lower respiratory tract and, besides common, rather mild symptoms, has been related to viral pneumonia and exacerbated COPD [26,30,103]. However, COVID-19, especially, caused by the novel SARS-CoV-2, displays a large variety of progress of disease, ranging from asymptomatic or flu-like symptoms to life-threatening and fatal outcomes, while also bearing the risk of various long-term effects [104,105]. While there were no approved vaccines against previous CoV variants, the SARS-CoV-2 pandemic accelerated vaccine development [102] and led to the first approval of an mRNA vaccine in history, at the end of 2020.

Recent studies have shown that CoV has a strong impact on the lipid metabolism in vitro [85,106]. Twenty-four different lipids, particularly lysophospholipids and fatty acids, were consistently upregulated in infected cells, as detected after infection with HCoV-229E or MERS-CoV. The linoleic acid (LA) to arachidonic acid (AA) metabolism axis was found to be especially perturbed, suggesting that this part of the lipid metabolism is significantly involved in CoV replication. Interestingly, the addition of exogenous LA and AA inhibited viral replication. The authors hypothesized that the supplementation may elicit a feedback reversion of lysophospholipids into phospholipids, hence disrupting the sensitive balance of metabolites [106]. Consistently, the inhibition of the cellular sterol regulatory element binding protein (SREBP), an important host factor for biosynthesis, intracellular movement and homeostasis of lipids, strongly decreased MERS-CoV and SARS-CoV propagation in various cell lines and MERS-CoV propagation, even in vivo [85]. A recent study (not yet peer-reviewed) showed reduced replication of SARS-CoV-2 after inhibition of various proteins of lipid metabolism, such as the PI3 kinase VPS34 by PIK-III or IN-I, lipases and FASN by Orlistat and long chain acyl-CoA synthetase by Triacsin C [107]. The high dependency of CoV on reshaping the cellular lipidome can be explained by the following facts: (i) the most likely reason is that CoV rearranges cellular membranes to form double-membrane vesicles (DMV) that serve as anchor structures for the viral replication/transcription complex and thus are crucial for successful replication [108,109,110]. The importance of lipid remodeling and DMV formation was highlighted by the observation that inhibition of the essential enzyme involved in these processes, the cytosolic phospholipase A_2_α, reduced CoV propagation [109]. Furthermore, (ii) lipids can provide energy for viral replication and play vital roles in the trafficking of viral components, as well as in the assembly and budding of new virus particles [106]. As it seems to be common for viruses in general, optimal CoV replication apparently has a requirement for a specific composition of the cellular lipidome.

A recent study, conducted with a SARS-CoV-2 isolate in human colorectal adenocarcinoma cells (Caco-2), reported reprogramming of several central cellular pathways, such as the central carbon (mainly glycolysis) and nucleic acid metabolism, splicing, translation and protein homeostasis [111]. Among others, proteins of metabolic pathways like carbon metabolism, the TCA cycle and the respiratory electron transport were upregulated during infection. Furthermore, various nucleic acid metabolic processes were shown to be correlated with SARS-CoV-2 protein expression. Accordingly, the inhibition of the afore-mentioned pathways (e.g., 2-DG to inhibit glycolysis or the guanosine analog ribavirin to impair nucleic acid synthesis) severely reduced viral replication, demonstrating the importance of these pathways for successful virus propagation [111]. In agreement with elevated levels of diverse metabolic proteins, the PI3K/AKT/mTOR signaling pathway was found to be stimulated during MERS–CoV infections. Inhibition of this important modulator of metabolism and other processes resulted in reduced viral replication [112]. Very recent in vitro data (not yet peer reviewed) showed an upregulation of glucose transporters and higher levels of several glycolytic enzymes in SARS-CoV-2-infected cells, as well as a strong reduction of viral multiplication and a lower infectivity of progeny particles after treatment with 2-DG. The authors of this study attributed these findings to the reversion of the virus-induced anabolic state and the strongly reduced glycosylation of viral envelope proteins [113]. Concurrently, an in silico study has proposed that 2-DG binds and thereby inactivates the important SARS-CoV-2 protease 3CLpro and the endoribonuclease NSP15, hence mitigating SARS-CoV-2 infections [114]. This would assign 2-DG an antiviral property other than metabolic interference.

Another recent in vitro SARS-CoV-2 study (not yet peer reviewed) describes deregulation of the PPP upon infection and antiviral effects after inhibition of the PPP by benfooxythiamine. This antiviral effect even increased in combination with 2-DG [115]. Besides, diverse nucleotide and nucleoside analogue inhibitors have already displayed varying effectivity in inhibiting the replication of known CoV strains [116]. The modes of action of this type of inhibitor can be the early termination of nucleic acid synthesis, depletion of the respective nucleotide pool and consequential inhibition of viral RNA synthesis or the induction of non-viable mutagenesis after the incorporation of the analogue’s triphosphate form by the viral polymerase [117,118].

In compliance with the close connection of CoV infections and host metabolism, metabolic disorders like type-2 diabetes, obesity or hypertension constitute considerable risk factors of COVID-19 and other severe CoV infections [119,120]. Altogether, CoVs seem to rely mainly on glucose, and especially lipid, metabolism for their replication. In this context the SARS-CoV-2 pandemic boosted research on metabolic antiviral host targets, which resulted in a multitude of potential candidates. While interference with many of these candidate targets already showed promising results in vitro and/or in vivo, there are even more potential targets that have been proposed, but not yet experimentally tested [121].

## 7. Adenoviruses

AdVs comprise a group of non-enveloped, icosahedral viruses with a double-stranded DNA (dsDNA) genome, which belong to the family of *Adenoviridae*. As of today, there are more than 100 known types of human AdV (HAdV), which are subdivided into seven species, A–G [122]. The species B, C and E replicate preferably in the upper and lower respiratory tract, while the others normally infect gastrointestinal or renal cells, or the conjunctiva of the eyes [123]. Usually, HAdV infections cause mild infections and are common among persons of all age, but severe manifestations of respiratory infections have been linked to pneumonia, bronchitis and exacerbations of COPD [26,30,124]. Apart from a nucleoside analogue, Cidofovir, there is no approved antiviral drug treatment available [123,125]. Moreover, even though an oral vaccine used by the U.S. military proved to be efficient, no vaccine has been licensed, so far [124,126].

As early as in the 1950s, HAdV infections were known to elicit an increased uptake of glucose and an upregulation of glycolysis in infected cells, with a higher production of lactic acid, mimicking the Warburg effect [127,128]. A functioning TCA cycle was also found to be crucial for proper viral replication [129], while oxygen consumption rates decrease during infections [130]. More recently, a major impact of HAdV on the host cell’s glucose and glutamine metabolism, with a varying dependency of the virus on glutamine in different cell lines, was described [74,130,131]. Thai et al. [130] showed that the viral gene product E4ORF1 enters the nucleus, binds the transcription factor MYC, thereby enhances MYC-induced expression of glycolytic key enzymes, such as hexokinase 2 and phosphofructokinase 1, and thus increases glycolysis. Furthermore, the authors elucidated that a large fraction of the enhanced glycolytic carbon flux is directed towards the PPP and the production of nucleotides for increased nucleic acid synthesis for the generation of progeny virions. Consistently, the mRNA expression of certain PPP enzymes was significantly increased [130]. A four-fold increase of PPP activity upon HAdV infection reported in another study substantiated these observations [132]. Valdés et al. [133] analyzed, in detail, in which phase of the infection the observed changes were most pronounced. Among the highlighted findings were the overrepresentation of purine and pyriminidine biosynthesis, glycolysis and cytoskeleton regulatory pathways during the early phase of infection and an increasing activation of the PPP and MYC with the progression of infection [133]. Additionally, Thai et al. [74] discovered that E4ORF1-induced activation of MYC also influences the glutamine metabolism on several levels and increases glutaminolysis and its intermediates in general. By the upregulation of glutamine transporters, the uptake of glutamine was increased. Moreover, the utilization of glutamine for the synthesis of intermediates of the HBP and certain amino acids, as well as the reductive metabolization of glutamine to potentially serve as a citrate source, was increased in infected cells [74]. The fact that the knockdown and inhibition of glutaminase, a central enzyme of glutaminolysis, clearly reduced viral replication underlines the value of glutamine metabolism for HAdV propagation [74]. Interestingly and counterintuitively, inhibition of glycolysis via 2-DG or deprivation of glucose was beneficial for HAdV propagation in different cell lines in vitro and in an in vivo mouse model, while glutamine metabolism was essential for the virus [134]. This is an excellent example of how diverse metabolic needs of different viruses can be, since most other viruses categorically depend on cellular glycolysis.

Another study reported an increase of cellular lipid metabolism in HAdV-infected cells [135]. As the lipids were not incorporated into virus particles and even UV-inactivated viruses of the same type elicited a similar effect on the host’s lipid metabolism, the authors hypothesized that a structural viral component mediated the upregulation of lipids [135]. Along that line, an enhanced conversion of acetyl-CoA, which is required for lipid synthesis, from citrate was described [132]. Since AdV is not enveloped and its replication compartments are within the nucleus and do not require membranous structures [136], the reason for its upregulation of lipid metabolism is still unknown. Possibly some of the manifold signaling properties of certain lipids [137,138] are exploited by the virus. Unlike the previously mentioned metabolic pathways, cellular respiration was found to be clearly reduced upon infection with HAdV [130], further reflecting the features of the Warburg effect.

Taken together, adenoviral alterations of the host metabolism display some intriguing features, such as the essential role of the glutamine metabolism while glycolysis seems to be negligible or even disadvantageous for replication as well as the as yet unexplainable upregulation of lipid metabolism.

## 8. Therapeutic Potential of Metabolic Interference—What It Is and What It Might Become?

Most studies that assess the therapeutic usability of metabolic interference to counteract viral infections, and more precisely respiratory viruses, have been conducted in vitro. Various groups demonstrated decreased replication of RSV and PIV [42], RV [17], IV [22,75] and CoV [111,113] by inhibiting glucose metabolism. The discussed results suggest that especially IV depends on glycolysis during its replication, which is demonstrated by almost completely diminished viral spread after the respective treatment [75]. Inhibition was achieved by either impairing glycolysis directly with 2-DG (inhibition of hexokinase and glucose-6-phosphate isomerase [139,140]) and 3-bromopyruvate (inhibition of hexokinase [140,141]) or by the inhibition of the important regulatory PI3K/mTOR signaling pathway with BEZ235 [22] in diverse immortalized and primary cell lines. Additionally, the suppression of HIF-1α by PX-478 decreased RSV replication [20]. Apart from glucose metabolism, Thai et al. [74] demonstrated a reduced propagation of IAV, and especially of AdV, in primary NHBE cells by inhibiting glutaminase and consequently glutaminolysis via the inhibitor CB-839. In addition to virus-restricting effects by inhibition of glucose metabolism or glutaminolysis, inhibition of certain events of lipid metabolism, often FAS, seems to have the broadest antiviral effect of the here-mentioned experimental treatments. The replication of RSV [49], RV [49,53], IV [83,84,85], PIV [49], CoV [85] and AdV [85] can evidently be reduced by, among others, the inhibition of FASN with TVB-3166 or C75 and the inhibition of acetyl-CoA carboxylase with TOFA in various immortalized cell lines or human bronchial epithelial cells—but, also, the inhibition of mere fatty acid import [84] or reduction of SREBP interactions and thus broad lipogenic gene expression with AM580 [85] clearly impaired viral replication. Focusing on nucleic acid metabolism, the PPP inhibitor benfooxythiamine and nucleoside analogue inhibitors such as the broad antiviral compound ribavirin decreased replication of CoV, in vitro [111,115,116], by interfering with the nucleotide metabolism. Besides which, ribavirin was already in use to treat diverse viral infections such as hepatits C virus [142], IV [143], RSV [144] and AdV [145], but, however, also displayed controversial results and is not recommended for routinely administration [144]. Finally, a study regarding the in vitro inhibition of OXPHOS, more precisely of cytochrome c reductase by antimycin A, showed strongly reduced titers of IAV [89]. Noteworthily, many of the antiviral effects of the above-mentioned metabolic treatments are not restricted to respiratory viruses but apply to viruses in general. For example, inhibition of glycolysis, glutaminolysis and/or FAS reduces replication or infectivity of, among others, human cytomegalovirus [83,146,147], herpes simplex virus [74,146,148], norovirus [149], HCV [117], varicella-zoster virus [150], dengue virus [151], West Nile virus [151], yellow fever virus [151], vaccinia virus [152,153], rotavirus [154] and poliovirus [155].

While there is a multitude of in vitro data on this topic, there are less reports containing results generated in vivo, which are more meaningful for the assessment of the usability of a potential therapeutic agent. Still, several studies have confirmed antiviral effects of the afore-mentioned compounds in in vivo models, most importantly without eliciting visible adverse effects. By using 2-DG in chick embryo [76] and mice [17], the replication of IV or RV, respectively, could be restricted efficiently. Furthermore, intranasal administration of 2-DG mitigated RV-induced inflammation in murine lungs [17]. Several other in vivo studies support the safety of 2-DG [156,157,158]. Additionally, the oral administration of BEZ235 and the pyruvate dehydrogenase kinase 4 inhibitor DADA increased survival rates of IAV-infected mice, reduced viral titers in their lungs, improved metabolic disturbances and suppressed cytokine storms [22,78]. The oral administration of the FASN inhibitor TVB-3166 achieved promising preventive and therapeutic results by reducing RSV replication in a mouse model and, at the same time, displayed less toxicity than the approved control compound, ribavirin, which strongly reduced RSV titers, as well [49]. Yuan et al. [85] demonstrated a clear antiviral effect of intraperitoneally injected AM580 in two different mouse lines against MERS-CoV and a highly pathogenic IAV strain, without showing any signs of toxicity. The treated mice exhibited lower viral loads, reduced loss of weight, increased survival and diminished alveolar tissue damage and inflammation [85]. Summarizing, the here-mentioned in vivo studies emphasize the possible usability of metabolic interventions as an antiviral strategy. However, there exist conflicting in vivo data, which also must be considered for evaluating the suitability of these methods as antiviral approaches. Wang et al. [159], for instance, reported 2-DG treatment in IAV-infected mice to be lethal. Possibly, these conflicting results can be attributed to different dosing of 2-DG, the specific virus subtype used or a higher susceptibility of the chosen mouse line to the compound. Nevertheless, it definitely demonstrates the complexity of this topic.

In terms of clinical trials, assessing the usability of any of the studied inhibitors to fight viral infections, the availability of data is scarce. Only few clinical trials with metabolic inhibitors were conducted in respect to viral infections, but, in the light of cancer research. Nevertheless these cancer studies could still provide information on the safety of a compound in general. However, regarding antiviral treatments and potential adverse effects, one has to consider that such a therapy would require only a short-term administration of a medication. The adverse effects observed in long-term antitumor studies may not occur or be milder in an antiviral treatment. Two clinical trials assessed the safety and tolerability of 2-DG in patients with diverse types of tumors. None of the tested doses of 2-DG met the maximum tolerated dose criteria [160] or changed vital parameters [161], and thus were found suitable. For the greater part, only mildly adverse effects, which resembled hypoglycemia, were observed [160,161]. The same hypoglycemia-like mild symptoms were also documented by others [162]. These data, combined with its broad antiviral activity and high tolerability in vitro and in vivo, plus the fact that 2-DG-mediated effects are reversible, makes it a promising candidate for further clinical studies. In the ongoing corona pandemic, such a phase III clinical trial is currently being conducted in India, which aims to evaluate the efficiency and safety of 2-DG as a treatment in combination with standard of care against acute SARS-CoV-2 infections (Clinical Trial Registry of India: CTRI/2021/01/030231). In addition to the potential clinical development of 2-DG, the safety and efficiency of the PI3K/mTOR inhibitor BEZ235 (also known as RTB101 or Dactolisib) was also assessed in several clinical trials. While two phase II cancer studies reported the poor tolerability of relatively high doses [163,164], others could show, in phase IIb and phase III trials and with the aim of finding therapies to improve immune functions in the elderly, that BEZ235 is well-tolerated at regular low doses [165]. Moreover, they indeed observed an increased IFN-mediated antiviral immune responses in the elderly [165]. While the authors could not statistically verify a drug-induced decrease in frequency or severity of viral respiratory infection, they also did not mean to exclude it. The verification of this correlation by further studies could render BEZ235 a clinically proven broad antiviral compound. In general, the just-mentioned types of clinical trials may determine if metabolic interference could become a licensed approach to treat severe viral infections in the future.

Since the idea to use metabolic interference as an antiviral treatment has only re-emerged a short while ago, there are too few clinical trials in this field, which are, however, crucial to validate possible future therapies. The potential of the here-summarized agents is huge, which is demonstrated by the manifold of positive in vitro and in vivo data. Of course, metabolic interference involves certain risks and problems: targeting metabolic pathways, which are essential for the host, has limitations, as such treatment should not have adverse effects that would worsen a patient’s conditions after infection. In addition, several studies have shown that the pattern of metabolic alterations of the same virus or its dependency on a specific metabolite/pathway may vary between different cell types, which makes it more complex to define a promising metabolic host target to restrict viral spread. Moreover, the efficiency of interfering with one metabolic pathway to mitigate infection differs between virus species. Even more complicating, to what extent the metabolic changes of infected cells can be attributed to the induction of the host cell immune response, instead of direct viral actions, remains largely elusive. While the production of cytokines can be very energy-demanding, some inflammatory mediators can directly modify cellular metabolism [91,96]. Hence, one should not underestimate the impact of the immune response on the host metabolism nor the interplay of these two domains, reviewed in greater detail by others [90,96]. Beside these complications, most studies have been conducted in vitro, and in vivo results or clinical trials, which would give us very important insights, could drastically diverge from them. Furthermore, for a medication to act as directed as possible in the target tissue and to minimize unintended effects elsewhere in the body, the mode of administration and the pharmacokinetics of the compound play important roles. On the other hand, targeting cellular factors or pathways implies the great advantage of an extremely high barrier for a pathogen to develop a resistance against the respective treatment. Since many viruses evolve rapidly, the steady development of drugs targeting viral factors seems to be a never-ending task. The benefits of metabolic interference have the potential to surpass its disadvantages once we have a better understanding of all the involved processes and can refine therapies therewith. Thus, the next step for research in the field of infection medicine should be to promote and to get involved in more clinical trials to tap the full potential of metabolic interference for curing severe viral infections. Moreover, as some studies have proved, metabolic interference is not only the one-dimensional inhibition of a single important enzyme but can also be accomplished and refined by interfering with associated cell signals, as is already common practice in treating diseases other than viral infection.

## 9. Conclusions

As obligate intracellular parasites, all viruses severely depend on the metabolisms of their host cells. Metabolic pathways, which are frequently affected by many viruses, are glycolysis, glutaminolysis, lipid metabolism, the PPP, nucleotide synthesis, the HBP, the TCA cycle and cellular respiration. Among these pathways, glycolysis and lipid metabolism appear to be the most promising candidates in terms of broad antiviral host targets. All of the here-mentioned respiratory viruses were demonstrated to be negatively affected by interference with these two pathways—with the exception of AdVs, which were unaffected by the inhibition of glycolysis. Furthermore, interfering with glutaminolysis, the PPP, OXPHOS or glycosylation of viral proteins was shown to impair replication of at least one of the listed viruses, thus, they also represent auspicious antiviral host targets. Interestingly, a shared feature of viral infections is the establishment of an overall anabolic state of the host cell to promote the generation of biomass to facilitate efficient production of new virions.

Our up-to-date knowledge suggests that different viruses induce different, but very specific reprogramming patterns of the cellular metabolism to create the environment they require for their individual, optimal replication. Unfortunately, most of the mechanisms by which viruses mediate such reprogramming still need to be unraveled. It has already been demonstrated that the direct activation, induction or stabilization of various metabolic mediators are mechanisms by which some viruses achieve metabolic modifications [20,22,70,71,72]. It is feasible that viruses, in general, exploit similar mechanisms. Therefore, targeting the cellular metabolism, or more precisely virus-induced metabolic alterations, represents a potential treatment against severe viral infections. To progress in this field it is essential to analyze the specific virus–host interactions, determine potential metabolic targets and evaluate the efficiency of treatments against the respective viruses, as well as their tolerability in the host.

Moreover, a more profound understanding of virus-induced metabolic changes and potential related therapies may also be of interest for other fields of research. For example, to a certain degree, the metabolic profile of virus-infected cells resembles that of tumor cells and, just like in tumor cells, various viruses were demonstrated to induce the Warburg effect in their host cells. In addition, several viruses were evidentially able to trigger oncogenesis through the changes they induced in their hosts [166,167]. Today, a growing number of scientists consider metabolic mutations and alterations not only to be a side effect of tumor transformation but possibly a kind of prerequisite for transformation and its progression [41,168]. Thus, it seems possible that the research fields of oncology and virology may further benefit from each other’s scientific discoveries. Nevertheless, many open questions remain to be answered to drive forward the progress in the interesting and promising field of metabolic virus-host interactions.

## Data Availability

No new data were created or analyzed in this study. Data sharing is not applicable to this study.
